# First record of *Phlebotomus sergenti* (Diptera: Psychodidae) in China and its potential epidemiological implications

**DOI:** 10.1371/journal.pntd.0014726

**Published:** 2026-10-05

**Authors:** Haowei Dong, Anjie Yang, Wenqi Shan, Chunyan Zhao, Xiangyu Li, Bing Rui, Jiangshan Zhao, Maimaitijiang Wumaier, Heng Peng, Yajun Ma

**Affiliations:** 1 Department of Pathogen Biology, College of Basic Medical Sciences, Naval Medical University, Shanghai, China; 2 Key Laboratory of Biosafety Defense, Ministry of Education, Shanghai, China; 3 Faculty of Naval Medicine, Naval Medical University, Shanghai, China; 4 Institute of Parasitic and Brucellosis Prevention and Treatment, Center for Disease Control and Prevention of Xinjiang Uygur Autonomous Region, Urumqi, China; University of Bari, ITALY

## Abstract

**Background:**

Leishmaniasis is a severe parasitic disease transmitted by sand fly bites. *Phlebotomus sergenti* is a principal vector of *Leishmania tropica*, causing anthroponotic cutaneous leishmaniasis (ACL) across the Mediterranean, North Africa, South Asia, and West Asia. The Xinjiang Uygur Autonomous Region, located in northwestern China, is a well-recognized endemic focus of visceral leishmaniasis (VL), with sporadic cutaneous leishmaniasis cases also documented in the region. Nevertheless, *Ph. sergenti* has never been reported in China prior to this study.

**Methodology/principal findings:**

Field investigations were carried out in Artux City (July 2023) and Akto County (August–September 2024), Kizilsu Kirghiz Autonomous Prefecture, Xinjiang Uygur Autonomous Region. Sand flies were collected using light traps in livestock pens and residential areas. A total of 2,013 sand fly specimens were collected and 319 of them were identified through morphological examination of the pharynx, spermathecae, and genitalia, combined with molecular analysis of mitochondrial cytochrome c oxidase subunit I (COI) and cytochrome b (Cyt b) genes. Among the identified specimens, 145 specimens were identified as *Ph. sergenti* (36 from Artux City, 109 from Akto County), representing the first record of this species in China. Phylogenetic analysis based on COI and Cyt b sequences showed that Chinese *Ph. sergenti* populations clustered with those from neighboring countries (e.g., Afghanistan), with no significant genetic differentiation between the two sampling localities. Metagenomic screening of pooled samples revealed the presence of Wolbachia endosymbionts but no detection of *Leishmania* spp., *Trypanosoma* spp. or *Bartonella* spp. in the collected *Ph. sergenti* specimens.

**Conclusions/significance:**

This study represents the first confirmed record of *Ph. sergenti* in China, filling a critical gap in the distribution map of this medically important sand fly species. The occurrence of *Ph. sergenti* at the China—Kyrgyzstan border region suggests potential transboundary dispersal of this vector and underscores the need for enhanced surveillance and cross-border collaborative efforts to monitor the spread of leishmaniasis and other vector-borne diseases in Central Asia.

## Author summary

Leishmaniasis is a widespread parasitic disease transmitted by tiny sand flies, posing a persistent public health burden across many tropical and subtropical regions. In northwest China, Xinjiang has long been recognized as an endemic focus of severe visceral leishmaniasis, yet one key vector species, *Phlebotomus sergenti*, had never been documented in China prior to our study. We conducted field investigations in two border localities of Xinjiang in 2023 and 2024, collecting sand flies from livestock shelters and residential yards. Through combined morphological examination and DNA sequencing, we formally confirmed the presence of 145 *Ph. sergenti* specimens, representing the first record of this species in China. Our genetic analysis revealed close relationships between these Chinese populations and those from neighboring Central Asian countries, indicating potential transboundary dispersal of the vector. No *Leishmania* parasites were detected in the tested samples, whereas *Wolbachia* endosymbionts were widely present in the collected sand flies. Our findings fill a critical gap in the global distribution of this medically important insect. They also highlight the need for enhanced long-term surveillance and cross-border collaboration to guard against the potential spread of cutaneous leishmaniasis in the region.

## Introduction

Leishmaniasis remains a significant global public health concern, with an estimated 700,000–1 million new cases annually. Untreated visceral leishmaniasis (VL) is associated with a case fatality rate exceeding 95% [1]. Transmitted by the bites of infected phlebotomine sand flies (Diptera: Psychodidae), the disease displays marked geographical clustering, with the highest burden in Brazil, the Middle East, Central Asia, and countries bordering the Mediterranean Sea [1,2].

In China, leishmaniasis is mainly endemic in Xinjiang, Gansu, Sichuan, Shaanxi, Shanxi, and Inner Mongolia, accounting for over 90% of all reported cases [3–5]. According to epidemiological characteristics, Chinese leishmaniasis is categorized into three types: anthroponotic, zoonotic, and natural focal [6]. Xinjiang, a historically high-endemic region, was once dominated by anthroponotic and natural focal leishmaniasis, with 403 cases reported in 2015 (excluding Xinjiang Production and Construction Corps) [4]. Following the implementation of intensified control strategies, the incidence has dropped substantially, with fewer than 10 cases reported annually since 2019 [4]. To date, *Leishmania infantum* and *L. donovani* are recognized as the main causative agents in Xinjiang, and *Phlebotomus wui*, *Ph. longiductus*, and *Ph. alexandri* are confirmed local vector species [7,8].

*Phlebotomus sergenti* is a principal vector of Old World cutaneous leishmaniasis, with a wide distribution across arid and semi-arid regions of the Mediterranean, North Africa, South Asia, and West Asia, particularly between 32°N and 37°N latitude (e.g., Morocco, Saudi Arabia, Iran, and Pakistan) [9–11]. Its geographical distribution and seasonal dynamics are strongly modulated by temperature and altitude [10,12]. This species primarily transmits *L. tropica*, causing anthroponotic cutaneous leishmaniasis (ACL). In addition, *L. infantum* DNA has been detected in *Ph. sergenti* in some regions, suggesting its potential involvement in VL transmission [10,11,13].

With the advancement of the Belt and Road Initiative (BRI), growing economic and personnel exchanges between China and countries along the route have raised the risk of cross-border incursion of vector species [14,15]. To elucidate the phlebotomine sand fly fauna in the border regions of Xinjiang, we carried out field investigations in Artux City in 2023 and Akto County in 2024, both in Kizilsu Kirghiz Autonomous Prefecture. Here, we document the first record of *Ph. sergenti* in China, and provide a comprehensive characterization of its morphological and molecular features, together with an assessment of its potential epidemiological implications.

## Materials and methods

### Ethics statement

All field investigations and sand fly samplings were conducted with prior approval from local residents and relevant local authorities. The sand fly species collected in this study are not listed as endangered or protected species, and all sampling procedures complied with local regulations on biological resource collection.

### Sampling site characteristics

Sand fly sampling was conducted in July 2023 in Artux City and August—September 2024 in Akto County, both in Kizilsu Kirghiz Autonomous Prefecture, Xinjiang Uygur Autonomous Region, China (Fig 1). Artux City and Akto County are located at the westernmost edge of China, with Artux City bordering Kyrgyzstan. Situated at the western edge of the Tarim Basin in the interior of the Eurasian continent at mid-latitudes, the study region has a warm temperate continental arid climate, with mean annual temperatures of 11–13°C. Summers are hot and arid, with maximum temperatures exceeding 30°C, whereas winters are cold and harsh, with minimum temperatures below -10°C. The region has abundant sunshine, scarce rainfall, and high evaporation. Soils are predominantly composed of silt, sand and gravel, with generally low organic matter content, and the native vegetation is dominated by drought-tolerant shrubs of the genera *Tamarix* and *Alhagi*.

**Fig 1:**
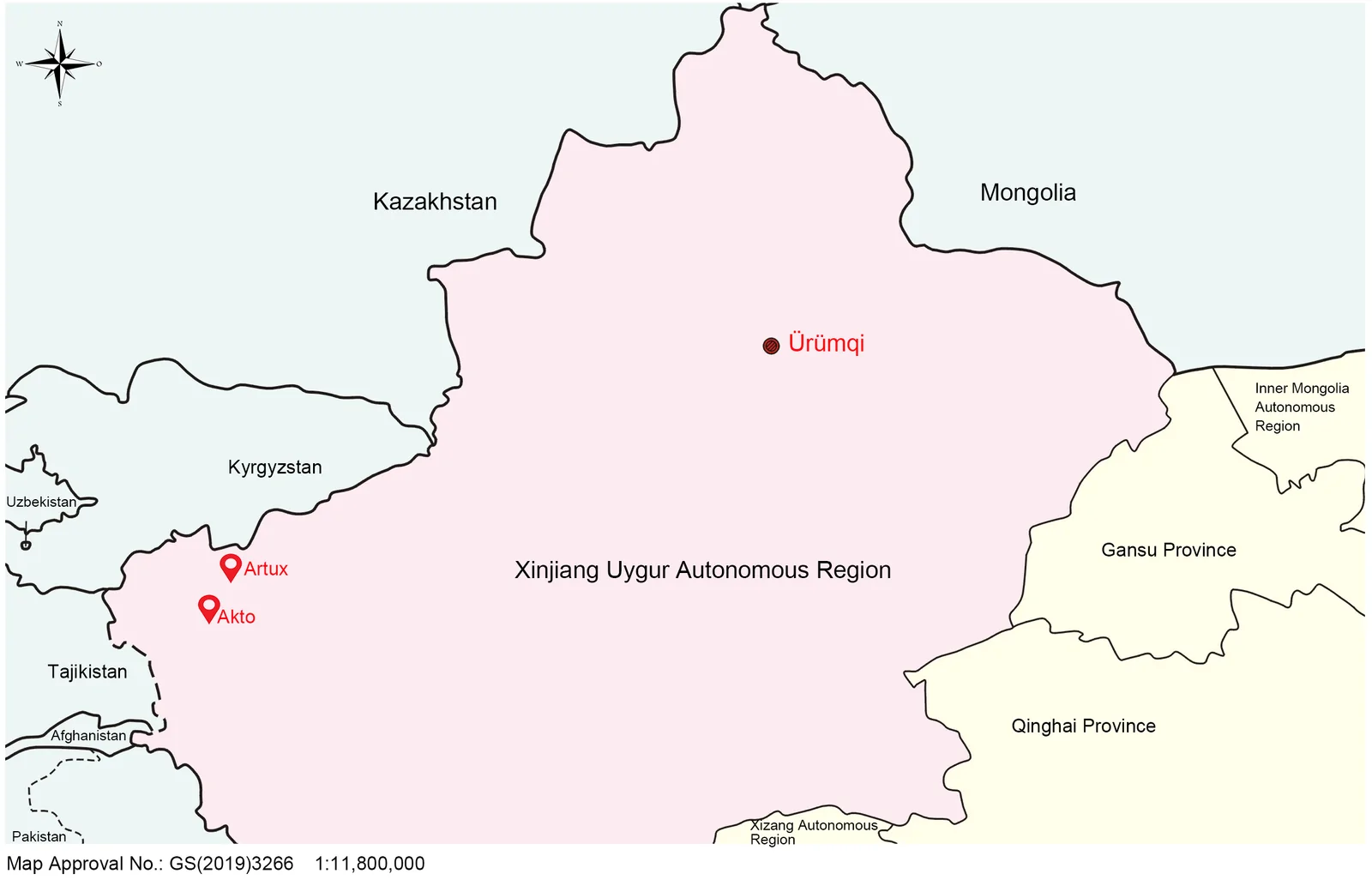
Geographic locations of the sand fly sampling sites. Red markers indicate the two sampling sites in Artux City and Akto County, Xinjiang Uygur Autonomous Region, China. Base map is the official standard map from the Standard Map Service System, Ministry of Natural Resources of China (Map review No. GS(2016)1600. No alterations were made to national boundaries on this base map.

Ahu Township (Artux City) is situated in the city’s northwestern area, while Baren Township(Akto County) lies on the eastern piedmont of the Kunlun Mountains, with both sampling sites located at elevations above 1,200 m (Table 1). Sampling was primarily carried out in enclosed and semi-enclosed sheep pens, which also reared large numbers of domestic pigeons and a small number of chickens. All pens had earthen floors with accumulated livestock manure. These livestock enclosures were situated in close proximity to residential dwellings, and the surrounding landscape consists of gravel desert with sparse vegetation. Additional sampling was performed inside residential dwellings and in their associated courtyards (Fig 2).

**Table 1:** Sand fly sampling information.

Collection site	Collection date	Environment	Sample size	Latitude/longitude	Altitude (m)
Artux City	Jul 2023	Livestock pen, human dwelling and courtyard	213	39.752° N, 76.065° E	1379
Akto County	Aug–Sep 2024	Livestock pen, human dwelling and courtyard	1800	39.158° N, 75.962° E	1257

**Fig 2:**
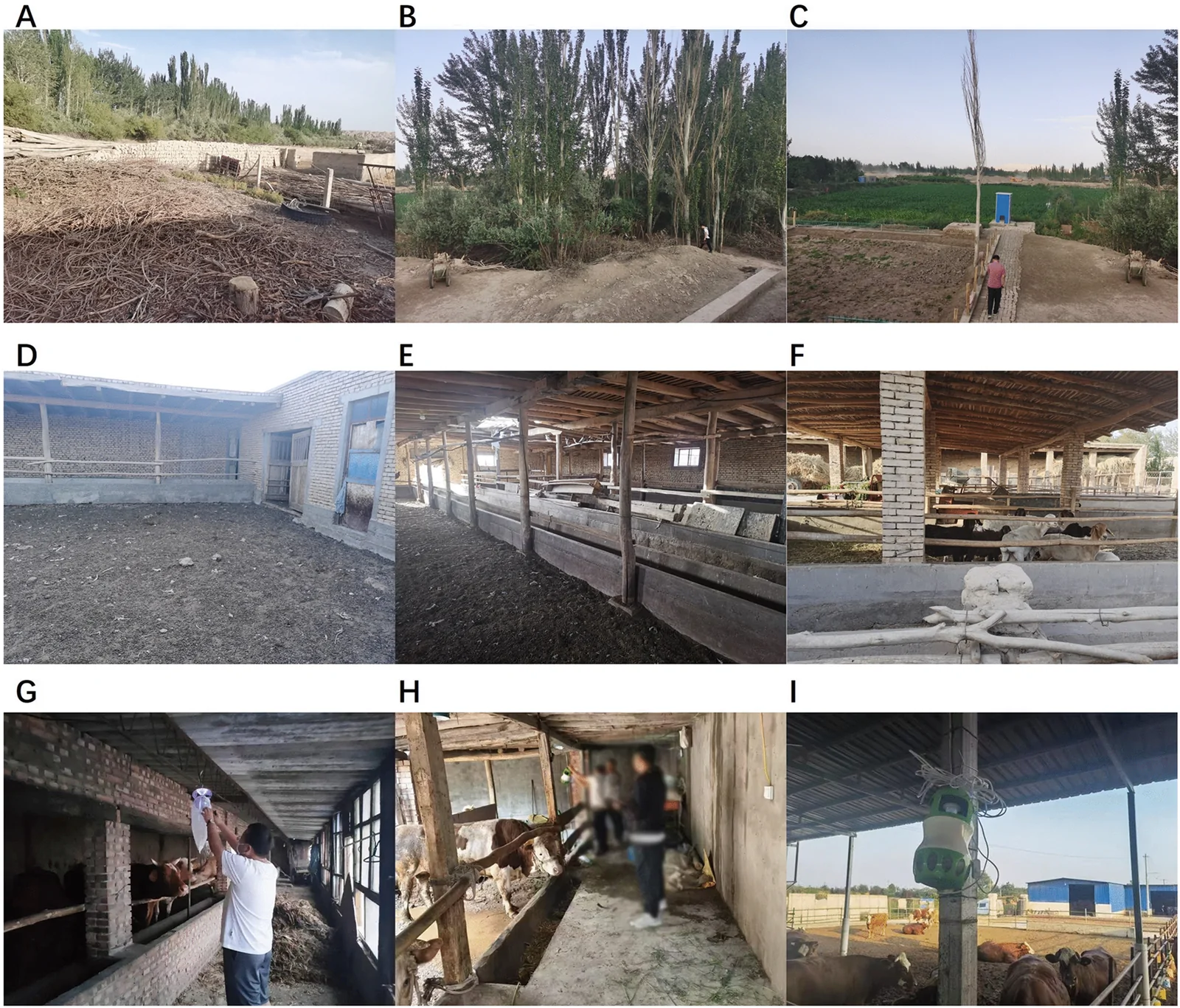
Representative habitats of the sand fly sampling sites. A–F: Field landscapes of sampling sites in Artux City; G–I: Field landscapes of sampling sites in Akto County.

### Sampling methods

Light traps (Model MYSF-HJY-1; Dongguan Houji Electronics Co., Ltd., China) were placed in livestock pens, residential courtyards, and poultry houses—habitats with documented high sand fly activity. Traps were suspended approximately 1.5 m above the ground in shaded, wind-protected areas. Sampling was carried out continuously from dusk to dawn, and traps were recovered the following morning. Captured sand flies were anesthetized with chloroform vapor. Intact adult specimens were sorted and pooled into batches of 10 individuals per tube. All samples were stored in 1.5 mL centrifuge tubes with silica gel desiccant, transported to the laboratory, and stored at −20°C until subsequent morphological and molecular analyses.

### Morphological identification

Individual sand flies were rinsed in 75% ethanol to remove surface debris, followed by three consecutive washes in sterile distilled water. The head and abdominal segments VIII–X were dissected from each specimen under a stereomicroscope (SZX10; Olympus Corporation, Tokyo, Japan) using sterile needles, and transferred to 0.2 mL PCR tubes with 10% potassium hydroxide (KOH) solution for tissue clearing and digestion at 37°C for 20 h. Following digestion, the dissected tissues were washed three times with sterile distilled water and mounted on glass slides. Taxonomic identification was conducted by examination of key diagnostic morphological characters, including the pharynx, mouthparts, and genitalia, in accordance with standard taxonomic keys [16]. Photomicrographs of diagnostic structures were taken using the microscope’s imaging system. The remaining body parts (thorax and anterior abdominal segments) of each specimen were individually stored in 1.5 mL centrifuge tubes, with a unique identifier linking each sample to its corresponding dissected tissues, and were used for subsequent DNA extraction.

### Molecular identification

The remaining body tissues (thorax and anterior abdominal segments) from each specimen were individually ground to a fine powder in liquid nitrogen using sterile disposable pestles. Total genomic DNA (gDNA) was extracted using the DNeasy Blood & Tissue Kit (Cat. No. 69504; Qiagen GmbH, Hilden, Germany) following the manufacturer’s protocol. Two mitochondrial gene fragments, mitochondrial cytochrome c oxidase subunit I (COI) and cytochrome b (Cyt b) gene fragments were amplified by PCR. Detailed primer sequences and thermal cycling conditions are summarized in S1 Table. PCR products were purified and subjected to Sanger sequencing. Raw sequences were assembled and quality- filtered using SeqMan (DNASTAR Inc., Madison, WI, USA). Consensus sequences were then aligned against reference sequences deposited in GenBank using BLAST analysis. Final species assignment was determined by cross-validating molecular identification results with the morphological identification data.

### Phylogenetic analysis

Reference sequences of the COI and Cyt b genes were retrieved from GenBank, including *Ph. sergenti* sequences from different geographical populations, other species belonging to the same subgenus, and *Ph. alexandri* as a reference taxon. Multiple sequence alignment was performed using the ClustalW algorithm implemented in MEGA XII [17]. The aligned sequences were trimmed to remove low-quality regions and ambiguous alignment sites. Pairwise genetic distances were then calculated based on the Kimura 2-parameter (K2P) substitution model. Maximum Likelihood (ML) phylogenetic trees were constructed using PhyloSuite v1.2.3 [18], with nodal support evaluated via 1000 bootstrap replicates. The final phylogenetic trees were visualized, annotated and polished using the tvBOT online platform (https://www.chiplot.online/tvbot.html) [19].

### Pathogen and symbiont detection

Following species identification, we selected 20 adult specimens per species (*Ph. sergenti*, *Ph. alexandri*, and *Ph. wui*) and pooled their genomic DNA separately for the construction of metagenomic sequencing libraries. DNA concentration and integrity were verified using a Qubit 4.0 fluorometer (Thermo Fisher Scientific, Waltham, MA, USA) and 1% agarose gel electrophoresis. For each library, 1 μg of qualified DNA was fragmented to ~350 bp using a Covaris M220 ultrasonicator (Covaris Inc., Woburn, MA, USA). Libraries were prepared using the Illumina TruSeq Nano DNA Library Prep Kit (Illumina Inc., San Diego, CA, USA), including end repair, A-tailing, adapter ligation, and 8–10 cycles of PCR enrichment. Final library quality was assessed with an Agilent 2100 Bioanalyzer (Agilent Technologies, Santa Clara, CA, USA) and Qubit 4.0. Qualified libraries were diluted to 2 nmol/L and sequenced on an Illumina NovaSeq 6000 platform with paired-end 150 bp reads.

Bioinformatics analysis was conducted according to the following pipeline. Trimmomatic v0.39 was used to filter reads containing adapters, low-quality reads (with <80% of bases achieving Q30 quality), or N bases ≥ 5% to obtain clean data. Clean reads were assembled de novo using MEGAHIT v1.2.9 (k-mer 21–141) to obtain contigs [20]. Open reading frames (ORFs) were predicted using Prodigal v2.6.3, and non-redundant gene sets were constructed via CD-HIT v4.8.1 (identity ≥ 95%, coverage ≥ 90%) [21,22]. Taxonomic profiling was carried out using MetaPhlAn 4.0 [23]. The presence of *Leishmania* spp. and other target vector-borne pathogens was verified by BLASTn (E-value ≤ 1e-5). Microbial relative abundance was quantified using Salmon v1.10.0, and differences in pathogen and symbiont community composition across the three sand fly species were subsequently analyzed [24].

## Results

### Sand fly sampling and morphological identification

A total of 2,013 sand fly specimens were collected across the two sampling sites, including 213 from Artux City and 1,800 from Akto County. Based on morphological examination of pharyngeal armature, spermathecae, and male genitalia, 319 specimens were identified and assigned to four species: *Ph. alexandri*, *Ph. longiductus*, *Ph. sergenti*, and *Ph. wui* (Fig 3).

**Fig 3:**
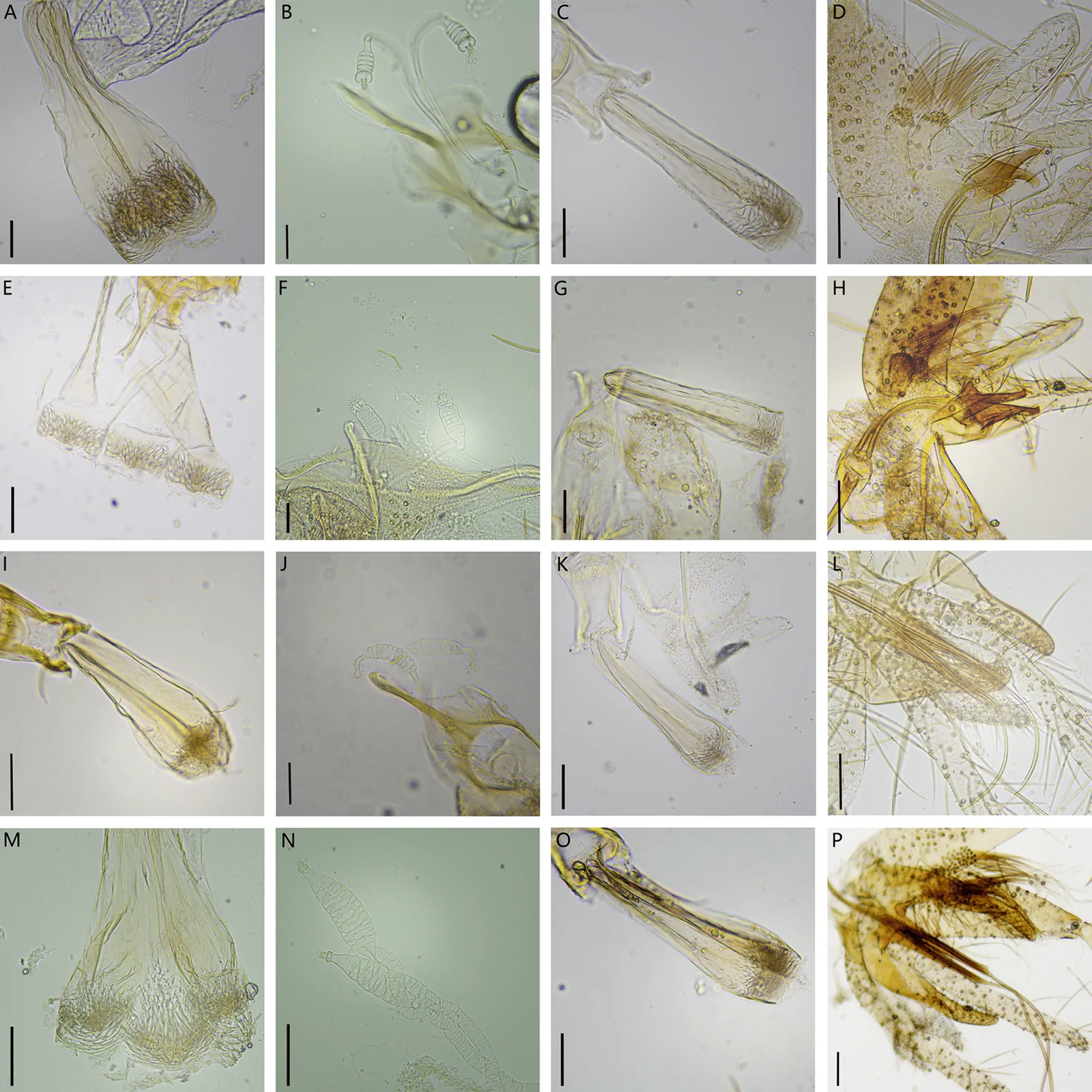
Diagnostic morphological characters of the dominant sand fly species in the study area. A–D: *Phlebotomus sergenti*; E–H: *Ph. alexandri*; I–L: *Ph. wui*; M–P: *Ph. longiductus*. Panels A, E, I, M show the female pharynx; B, F, J, N show the spermatheca; C, G, K, O show the male pharynx; D, H, L, P show the male genitalia. Scale bar = 50 μm for all micrographs.

In Artux City, 168 specimens were successfully identified, consisting of 91 *Ph. alexandri* (55 males, 36 females), 41 *Ph. wui* (2 males, 39 females), and 36 *Ph. sergenti* (19 males, 17 females). In Akto County, 151 specimens were identified, comprising 23 *Ph. alexandri* (all female), 19 *Ph. longiductus* (5 males, 14 females) and 109 *Ph. sergenti* (54 males, 55 females) (Table 2).

**Table 2:** Sand fly specimen identification results.

Collection site	Species	No. of morphological identification	No. of molecular identification	
			COI	Cyt b
Artux City	*Ph. alexandri*	91 (55♂, 36♀)	52	52
	*Ph. sergenti*	36 (19♂, 17♀)	31	44
	*Ph. wui*	41 (2♂, 39♀)	10	10
Akto County	*Ph. alexandri*	23 (0♂, 23♀)	5	5
	*Ph. longiductus*	19 (5♂, 14♀)	5	5
	*Ph. sergenti*	109 (54♂, 55♀)	8	2

Note: Only a subset of the collected specimens was subjected to morphological and molecular identification.

Notably, this finding constitutes the first formal record of *Ph. sergenti* in China. *Phlebotomus sergenti* belongs to the subgenus *Paraphlebotomus*, while *Ph. alexandri* has recently been reassigned to the subgenus *Artemievus* [25]. Despite belonging to different subgenera, the two species exhibit considerable morphological similarity, which can lead to misidentification in routine surveillance. The key diagnostic differences between them are summarized as follows: 1. *Phlebotomus sergenti* has an elongated pharyngeal armature with transverse ridges at the apex and uniform pigmentation. In contrast, *Ph. alexandri* has a short, wide pharyngeal armature, lacks apical transverse ridges, and displays uneven pigmentation. 2. The spermathecae of both *Ph. sergenti* and *Ph. alexandri* exhibit distinct segmentation. The apical spermathecal segment is enlarged in *Ph. sergenti*, whereas in *Ph. alexandri* this segment is the smallest of all segments. 3. The gonocoxite of *Ph. sergenti* bears a basally located, finger-shaped protuberance that curves distally. A homologous protuberance is present at the same position in *Ph. alexandri*, but it is shorter than that of *Ph. sergenti* with a rounded apex. Brush-like setae are present on the protuberance in both species. The setae of *Ph. alexandri* are long and stiff, whereas those of *Ph. sergenti* are slightly shorter and curved apically.

### Molecular identification results

COI and Cyt b gene sequences were successfully obtained for *Ph. sergenti*, *Ph. alexandri*, and *Ph. wui*. For Cyt b gene identification, a total of 118 individuals were successfully sequenced, including 106 from Artux City (44 *Ph. sergenti*, 52 *Ph. alexandri*, 10 *Ph. wui*) and 12 from Akto County (2 *Ph. sergenti*, 5 *Ph. alexandrii, 5 Ph. longiductus*). For COI gene identification, a total of 111 individuals were successfully sequenced, including 93 from Artux City (31 *Ph. sergenti*, 52 *Ph. alexandri*, 10 *Ph. wui*) and 18 from Akto County (8 *Ph. sergenti*, 5 *Ph. alexandri, 5 Ph. longiductus*). Species identity was confirmed via BLAST alignment, with the COI and Cyt b sequences of *Ph. sergenti* showing >95% nucleotide similarity to published reference sequences.

### Phylogenetic analysis

Phylogenetic trees were reconstructed based on COI (Fig 4) and Cyt b (Fig 5) sequences, with *Lutzomyia longipalpis* designated as the outgroup, and *Ph. chinensis* included as an internal reference taxon. The two trees showed highly consistent clustering patterns among ingroup taxa, with nearly all sequences forming distinct conspecific clusters, confirming the reliable species-level resolution of these mitochondrial markers for the majority of morphologically identified species.

**Fig 4:**
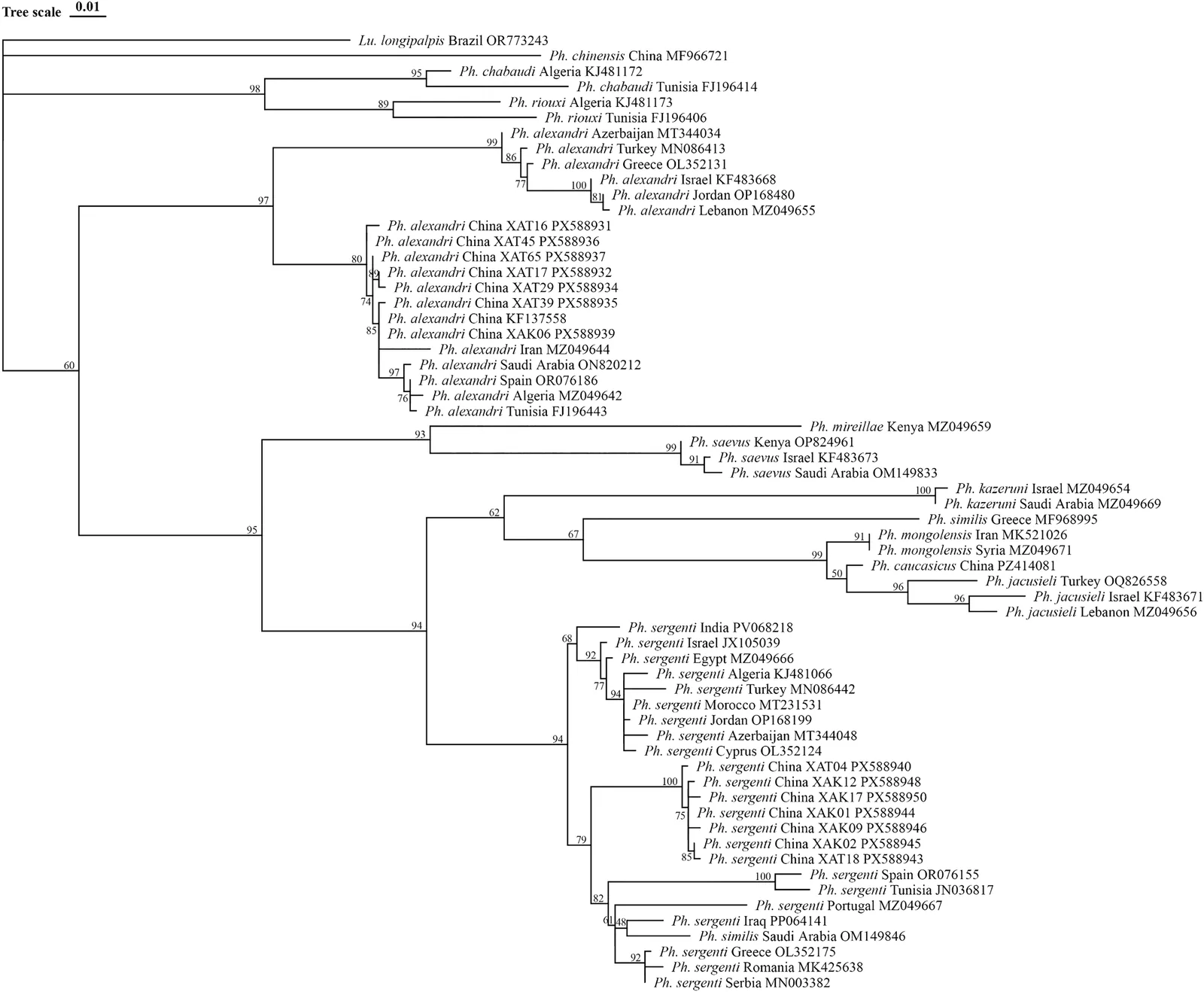
Maximum Likelihood phylogenetic trees of *Phlebotomus* species constructed from mitochondrial COI gene fragments. Bootstrap support values (1000 replicates) are indicated on branches. Scale bars represent genetic distances is 0.01.

**Fig 5:**
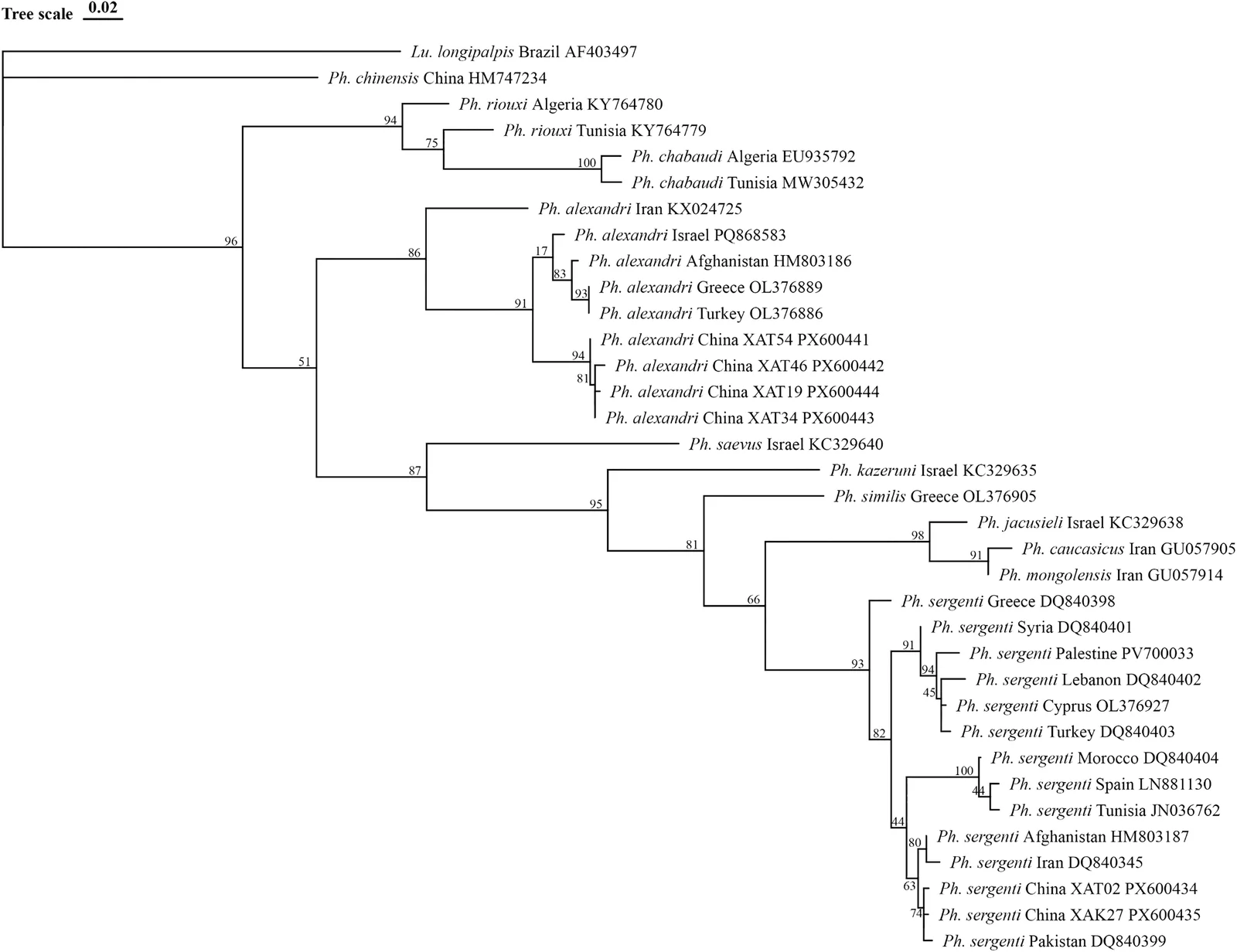
Maximum Likelihood phylogenetic trees of *Phlebotomus* species constructed from mitochondrial Cyt b gene fragments. Bootstrap support values (1000 replicates) are indicated on branches. Scale bars represent genetic distances is 0.02.

*Phlebotomus alexandri* formed a well-supported monophyletic clade in both trees and consistently occupied a basal position relative to the *Ph. sergenti* complex and its allied species. Regarding internal phylogeographic structure, the two markers differed in phylogenetic resolution: COI sequences from China did not form an independent geographic clade but were nested within lineages from the Mediterranean region and adjacent areas, whereas Cyt b sequences from China constituted a distinct and well-supported subclade sister to a group comprising samples from West Asia and the eastern Mediterranean. Deep internal nodes within *Ph. alexandri* received low bootstrap support in both datasets, indicating that neither COI nor Cyt b alone can fully resolve the recent radiation within this species.

*Phlebotomus sergenti* also formed a strongly supported monophyletic group in both analyses, with clear phylogeographic structure. The COI tree revealed that Chinese *Ph. sergenti* sequences formed a monophyletic subclade nested within Eurasian lineages, showing complex nested relationships with populations from other regions. All *Ph. sergenti* specimens from this study clustered within the species clade with no anomalous individuals. The Cyt b tree identified two major well-supported lineages within *Ph. sergenti*: one comprised samples from the eastern Mediterranean and the Levant region, and the other comprised samples spanning West Asia, North Africa, the western Mediterranean and East Asia.

In the COI tree, a single GenBank sequence annotated as *Ph. similis* (OM149846, Saudi Arabia) was nested within the *Ph. sergenti* clade, rendering *Ph. similis* polyphyletic. Given the high morphological similarity between the two species, this anomalous placement likely results from specimen misidentification and the taxonomic identity of this sequence requires further validation.

Comparison of the COI and Cyt b trees revealed partial conflict in the phylogeographic affinities of certain *Ph. sergenti* populations, indicating that different mitochondrial genes may record divergent evolutionary histories at the intraspecific level. Despite these minor incongruences, the two trees were concordant at higher taxonomic levels: the *Ph. sergenti* group consistently formed a sister relationship with a clade containing *Ph. caucasicus*, *Ph. mongolensis*, *Ph. saevus*, *Ph. kazeruni* and related species, while *Ph. alexandri* remained in a consistently more basal position. This stable topology supports the independent evolutionary trajectories of *Ph. alexandri* and the *Ph. sergenti* complex, representing two deeply divergent ancient lineages.

### Pathogen and symbiont detection

Metagenomic sequencing was performed on pooled samples of *Ph. sergenti*, *Ph. alexandri*, and *Ph. wui* from the two sampling sites to screen for vector-borne pathogens and bacterial symbionts. No genomic sequences of *Leishmania* spp., *Trypanosoma* spp. or *Bartonella* spp. were detected in any sample pool, indicating no evidence of these pathogens in the tested sand fly populations.

In contrast, *Wolbachia* endosymbionts were widely detected in samples from both Artux and Akto (Table 3). Across all four sample pools, sequences matching the *Wolbachia* endosymbiont of *Ceutorhynchus assimilis* were consistently the dominant microbial taxon. We note that this taxonomic assignment reflects the best-matched reference sequence (originally isolated from the weevil *C. assimilis*) and does not imply that sand flies carry weevil-derived symbionts. Definitive taxonomic classification of *Wolbachia* strains circulating in local sand fly populations will require further validation using multilocus sequence typing.

**Table 3:** Top 5 microbial abundance (species level) in sand fly samples.

Species-level abundance ranking	*Ph. sergenti* (Atux)	*Ph. sergenti* (Akto)	*Ph. alexandri* (Atux)	*Ph. wui* (Atux)
1	*Wolbachia endosymbiont of Ceutorhynchus assimilis*	*Wolbachia endosymbiont of Ceutorhynchus assimilis*	*Wolbachia endosymbiont of Ceutorhynchus assimilis*	*Wolbachia endosymbiont of Ceutorhynchus assimilis*
2	*Streptococcus pyogenes*	*Escherichia coli*	*Streptococcus pyogenes*	*Salpingoeca rosetta*
3	*Escherichia coli*	*Streptococcus pyogenes*	*Flavobacterium sp. JXAS1*	*Escherichia coli*
4	*Pseudomonas aeruginosa*	*Pseudomonas aeruginosa*	*Wolbachia endosymbiont of Psylliodes chrysocephala*	*Monosiga brevicollis*
5	*Solemya velum gill symbiont*	*Wolbachia endosymbiont of Psylliodes chrysocephala*	*Escherichia coli*	*Wolbachia endosymbiont of Psylliodes chrysocephala*

The species-level composition of the top five most abundant microbes in each sample pool is shown below: 1. *Phlebotomus sergenti* (Artux): *Wolbachia endosymbiont of Ceutorhynchus assimilis*, *Streptococcus pyogenes*, *Escherichia coli*, *Pseudomonas aeruginosa*, and *Solemya velum gill symbiont*; 2. *Phlebotomus sergenti* (Akto): *Wolbachia endosymbiont of Ceutorhynchus assimilis*, *Escherichia coli*, *Streptococcus pyogenes*, *Pseudomonas aeruginosa*, and *Wolbachia endosymbiont of Psylliodes chrysocephala*; 3. *Phlebotomus alexandri* (Artux): *Wolbachia endosymbiont of Ceutorhynchus assimilis*, *Streptococcus pyogenes*, *Flavobacterium sp.* JXAS1, *Wolbachia endosymbiont of Psylliodes chrysocephala*, and *Escherichia coli*; 4. *Phlebotomus wui* (Artux): *Wolbachia endosymbiont of Ceutorhynchus assimilis*, *Salpingoeca rosetta*, *Escherichia coli*, *Monosiga brevicollis*, and *Wolbachia endosymbiont of Psylliodes chrysocephala*.

## Discussion

This study reports the first confirmed record of *Ph. sergenti* in China through integrated morphological and molecular identification, filling a critical gap in the known geographical distribution of this medically important vector. Morphological examination revealed typical taxonomic characteristics of *Ph. sergenti*, including distinctly segmented female spermathecae with an enlarged apical segment, and a characteristic finger-shaped protuberance at the base of the gonocoxite in male genitalia. At the molecular level, phylogenetic trees based on the mitochondrial COI and Cyt b gene showed that sequences from this study clustered with *Ph. sergenti* populations from neighboring regions (Pakistan and Afghanistan), consistent with their geographical proximity, which further confirmed the reliability of species identification. Notably, all *Ph. sergenti* specimens in this study were collected from residential houses and livestock pens in western Xinjiang, indicating a close association with human-associated habitats and high potential for human–vector contact. This habitat preference is consistent with habitat preferences reported in Mediterranean and Central Asian regions.

*Phlebotomus sergenti* is one of the most widely distributed leishmaniasis vectors globally, and its vectorial importance has been extensively documented. It is the primary vector of *L. tropica*, the causative agent of anthroponotic cutaneous leishmaniasis, and plays a key role in disease epidemics in the Middle East and Mediterranean regions [10,26]. However, the epidemiological pattern of leishmaniasis in China differs markedly: most reported cases are visceral leishmaniasis caused by *L. donovani* and *L. infantum*, while cutaneous leishmaniasis has only been sporadically reported [4,5,8]. The Kizilsu Kirghiz Autonomous Prefecture reported 10 cases of visceral leishmaniasis from 2013–2023. Although metagenomic screening in this study did not detect *Leishmania* in the tested specimens, pathogen carriage rates may be influenced by seasonality and host density [27,28]. Given the complexity of leishmaniasis epidemiology in China, there is an urgent need to establish long-term surveillance systems to dynamically monitor pathogen carriage status and epidemic trends.

The newly recorded distribution of *Ph. sergenti* is located at the westernmost edge of China, bordering Kyrgyzstan. This geographical pattern provides critical evidence for transboundary dispersal of vector insects and disease prevention and control. The border areas of Xinjiang share high ecological similarity with Central Asia, and cross-border movement of people and livestock may facilitate transregional transmission of vector insects and their carried pathogens [29,30]. Against the background of the global resurgence of leishmaniasis, gaps in vector species distribution in border regions directly reduce the precision of control strategies. Therefore, cross-border collaborative surveys with neighboring countries are urgently needed to clarify geographic distribution boundaries and dispersal pathways of vectors, so as to provide scientific evidence for establishing regional leishmaniasis control barriers.

*Phlebotomus sergenti* has an extensive geographical range spanning the Mediterranean, Middle East, Central Asia, and South Asia, forming geographic populations adapted to different climatic and ecological conditions and showing significant genetic differentiation [10,31,32]. Molecular ecological studies based on COI, Cyt b, EF-1α gene and internal transcribed spacer (ITS) have shown that Mediterranean and Central Asian populations have reached critical values for population differentiation, which may be related to geographic isolation (e.g., mountains, deserts) and differences in host adaptation [33–36]. Differences in blood-feeding preference, fecundity, and pathogen transmission efficiency among different geographic populations have been confirmed. For example, Mediterranean populations show significantly higher anthropophilic tendencies than Central Asian steppe populations [10]. The genetic background of the newly recorded Chinese *Ph. sergenti* population and its association with neighboring West and South Asian populations, and its potential unique ecological adaptation characteristics remain to be further investigation with larger sample sizes and more comprehensive phylogenetic analysis. Such studies are critical for accurate assessment of vector-borne disease transmission risk posed by this species.

Intracellular symbionts, along with their community structure and function in vector insects, have become core targets for elucidating differences in vector competence and developing biological control strategies [37,38]. Recent studies have confirmed that *Ph. sergenti* carries various typical intracellular symbionts, such as *Wolbachia* and *Rickettsia* [39]. These symbionts can directly or indirectly affect the survival and development of *Leishmania* in vectors by regulating host immune responses, energy metabolism, and gene expression [40]. Although this study did not conduct in-depth analysis of the composition and genotyping of intracellular symbionts in *Ph. sergenti*, we characterized the dominant symbiont taxa, which lay a foundation for follow-up research. Future studies could combine metagenomic sequencing with fluorescence in situ hybridization to analyze the community structure and distribution characteristics of intracellular symbionts in Chinese *Ph. sergenti* populations, screen functional strains with *Leishmania* inhibitory activity, and explore the use of symbiont transfection technology to regulate vector competence. This research direction is of great significance for reducing reliance on chemical insecticide use and constructing eco-friendly control systems.

As the first report of *Ph. sergenti* distribution in China, this study inevitably has limitations that need to be addressed in future work. First, the survey scope only covered limited areas of western Xinjiang, and the distribution boundary of this species within China has not been clarified. Given its wide distribution in Central Asia, the possibility of distribution in other parts of Xinjiang regions and neighboring provinces such as Gansu and Inner Mongolia urgently requires investigation. Second, sampling was concentrated during peak sand fly activity season, lacking year-round dynamic monitoring data. Key biological traits such as overwintering strategy and number of generations could not be determined, and these characteristics directly shape seasonal dynamics of transmission risk. Additionally, this study only screened for protozoan and bacterial pathogens including *Leishmania*, *Trypanosoma* and *Bartonella*, and did not assess potential viral pathogens. The limited sample size may also have led to underestimation of pathogen carriage rates. Accordingly, future studies should expand the survey scope to the entire northwest arid and semi-arid region, establish cross-annual monitoring networks, and combine molecular detection with experimental infection experiments, systematically assess vector competence for different *Leishmania* species. Ultimately, scientific risk assessment models should be constructed to provide comprehensive support for formulating precise control strategies.

## Conclusion

This study provides the first formally confirmed record of *Phlebotomus sergenti* in mainland China, a medically significant phlebotomine sand fly and principal vector of Old World cutaneous leishmaniasis. We present full morphological and molecular characterization of this newly recorded population, and demonstrate its close phylogenetic affinity with conspecific populations from adjacent regions. These data fill a long-standing gap in the known distribution of *Ph. sergenti* and offer essential baseline information for studying the evolutionary dynamics of this vector across Central Asia. While no *Leishmania* pathogens were detected in the tested specimens, the widespread presence of *Wolbachia* endosymbionts indicates the complexity of tripartite interactions among vectors, pathogens and symbionts, which requires in-depth exploration. Our findings highlight the pressing need for enhanced long-term surveillance and cross-border cooperative initiatives to track the spread of leishmaniasis and other vector-borne diseases in Central Asia, especially as regional connectivity increases and climatic conditions continue to shift.

## Supporting information

S1 Table Primer sequences and thermal reaction parameters for amplification of the mitochondrial COI and Cyt b genes. (DOCX)
