# Supplementary material for: First record of Phlebotomus sergenti (Diptera: Psychodidae) in China and its potential epidemiological implications
Source: PLoS Negl Trop Dis. 2026 Oct 5;20(10):e0014726. doi: 10.1371/journal.pntd.0014726 (PMC13652333; doi:10.1371/journal.pntd.0014726)
Supplement: Supplementary file 1 [file pntd.0014726.s001.docx]

**S1 Table Primer sequences and PCR reaction conditions for COI and Cytb gene amplification**

| Marker | Name of primers | Primer sequences 5’-3’ | PCR reaction conditions | Size of the products in bp |
| --- | --- | --- | --- | --- |
| Mitochondrial COI | LepF | ATTCAACCAATCATAAAGATATTGG | 94℃ 2min, (94℃30s, 45℃ 30s, 72℃ 1min, 4 cycles) , (94℃ 30s, 49℃ 30s, 72℃ 1min, 35 cycles), 72℃ 8min | 709 |
|  | LepR | TAAACTTCTGGATGTCCAAAAAATCA |  |  |
| Mitochondrial Cytb | C3B-PDR | CATATTCAACCAGAATGATA | 94℃ 3min, (94℃30s, 40℃ 1min, 68℃ 1min, 5 cycles) , (94℃ 1min, 44℃ 1min, 68℃ 1min, 35 cycles), 68℃ 8min | 536 |
|  | N1N-PDR | GGTACATTGCCTCGAATTCGATATGA |  |  |
